# Association of hormone replacement therapy with mortality in colorectal cancer survivor: a systematic review and meta-analysis

**DOI:** 10.1186/s12885-019-6428-0

**Published:** 2019-12-09

**Authors:** Yeu-Chai Jang, Hsi-Lan Huang, Chi Yan Leung

**Affiliations:** 1 The Taipei Municipal Wanfang Hospital, Taipei, Taiwan; 2 Department of Global Health Policy, Graduate School of Medicine, The University of Tokyo, 7-3-1 Hongo, Bunkyo-ku, Tokyo, 113-0033 Japan; 3 Division of Cancer Statistics Integration, Center for Cancer Control and Information Services, National Cancer Center, Tokyo, Japan

**Keywords:** Hormone replacement therapy, Colorectal cancer survivor, Survival

## Abstract

**Background:**

Hormone replacement therapy (HRT) use has shown to be associated with a reduced risk of colorectal cancer, however, its impact on survival among women with colorectal cancer remains uncertain. This meta-analysis aimed to systematically assess the survival benefit of HRT use in patients with colorectal cancer.

**Methods:**

PRISMA guidelines for the reporting of meta-analyses were followed. We systematically searched PubMed, Embase, Cochrane library, Scopus, and PsycINFO from inception to 12 January 2019, with no language restrictions, for randomized controlled trials and cohort studies reporting the association between hormone replacement therapy and risk of colorectal cancer mortality or all-cause mortality in colorectal cancer survivors. We used the Newcastle-Ottawa Scale to assess the risk of bias of the included studies. We summarized the association as hazard ratio (HR; 95% CI) using random-effects meta-analysis. The study protocol was registered in PROSPERO (CRD42017071914).

**Results:**

Of 1648 articles identified, five cohorts including 10,013 colorectal cancer survivors were included in this meta-analysis. Compared with women with no prior use of HRT, those reporting current use of HRT had lower risks of colorectal cancer-specific mortality (HR, 0.71 [95% CI, 0.62–0.80], *I*^*2*^ = 0%) and overall mortality (HR, 0.74 [95% CI, 0.67–0.81], *I*^*2*^ = 0%). Low between-study variance was also suggested by the narrow prediction interval for colorectal cancer-specific mortality (0.58–0.86) and overall mortality (0.63–0.87), which indicated that a future study will show survival benefits in women with current HRT use compared with those with no HRT exposure. Inverse associations with colorectal cancer-specific (HR, 1.02 [95% CI, 0.82–1.28], *I*^*2*^ = 0%) and overall mortality (HR, 1.07 [95% CI, 0.90–1.27], *I*^*2*^ = 0%) were not observed for former users of HRT. Sensitivity analyses revealed no differences in the risk estimates between two groups.

**Conclusions:**

The findings suggest that the current use of HRT is associated with lower risks of colorectal cancer-specific and overall mortality in patients with colorectal cancer. Further investigations to elucidate the underlying mechanism are warranted.

## Background

Colorectal cancer is the third most common cause of cancer mortality in women, with 0.8 million new cases in 2018 worldwide [1]. The advances in treatment of colorectal cancer has translated to a marked improvement in survivorship in the past decades [1]. From 1995 to 2014, the 5-year survival was increased by 5–10% in various countries [2]. Given the aging population and advances in treatment in the past decades, the 5-year colorectal cancer prevalence in women were estimated to be over 2 million in 2018 [3]. Despite these encouraging figures, the cumulative impact of cancer and ongoing chronic physical and emotional symptoms reduce quality of life and overall survival [4].

Identification of factors associated with better prognosis among colorectal cancer survivors has important implications to inform provision of survivorship care. Extensive evidence from observational studies and clinical trials suggests that hormone replacement therapy (HRT) might have protective effect against colorectal cancer incidence. A meta-analysis of four randomized control trials and 16 observational studies showed a 26% reduction in colorectal cancer risk associated with any use of combined estrogen and progestin [5]. Similar association was observed for estrogen-only HRT despite considerable between-study heterogeneity [5]. However, previous evidence only focused on the association between HRT and colorectal cancer risk [5]. The role of HRT use on risk of mortality in patients diagnosed with colorectal cancer remains uncertain. A comprehensive evaluation of the impact of hormone therapy use among cancer survivors is urgently needed to inform physicians and patients for treatment decision making.

In this systematic review and meta-analysis, we aimed to summarize the available evidence and to quantify the association of HRT with colorectal-specific and all-cause mortality in women with diagnosis of colorectal cancer.

## Methods

### Search strategy and selection criteria

The review protocol was registered in PROSPERO (CRD42017071914). In this study, we followed Preferred Reporting Items for Systematic Reviews and Meta-Analyses (PRISMA) guidelines for the reporting of meta-analyses (See Additional file 1: Table S1) [6]. We systematically searched PubMed, Embase, Cochrane library, Scopus, and PsycINFO from inception to 12 January 2019, with no language restrictions, reporting the association between hormone replacement therapy and risk of colorectal cancer mortality or all-cause mortality in colorectal cancer survivors. Articles were searched using keywords and Mesh terms relating to hormone replacement therapy; and colorectal, colon, or rectal cancer survivor. In addition, we searched reference lists of identified articles (Details of search strategies are described in Additional file 1: Tables S2, S3, S4, S5 and S6).

We did this systematic review and meta-analysis according to prespecified methodological criteria: (1) Published original articles of randomized controlled trials or cohort studies with a minimum sample sizes larger than 50 participants; (2) Studies that enrolled colorectal cancer survivors aged 40 to 75 years, or studies that evaluated peri- or post-menopausal women; and (3) The exposure of interest was HRT. In the meta-analysis, study must either provide hazard ratio (HR), relative risk (RR), or odds ratio (OR) with 95% confidence intervals (CIs); or provide sufficient data that would allow the risk estimate to be calculated. The primary outcome was colorectal cancer-specific mortality. Secondary outcome was all-cause mortality in survivor with colorectal cancer. We excluded case-control studies, reviews, editorials, letters, and animal studies. For publications assessing duplicate population, only studies with larger sample size were included. For non-English articles, we consulted native speakers for translation. Two reviewers (YCJ and HLH) independently screened the title and abstract of potentially eligible articles for inclusion. Disagreement on eligibility was resolved by discussion between the reviewers.

### Data extraction and quality assessment

Two independent reviewers (YCJ and HLH) extracted the data from identified articles, and a third reviewer (CYL) crosschecked the abstracted data for accuracy. We extracted data using a standardized observation form, items of the form included name of first author, publication year, age, study design, study period, country; number of participants; cancer stage and grade at diagnosis; HRT type and recency; mortality (colorectal cancer and all-cause); and follow-up time. We extracted the most finely adjusted risk estimates from the included studies. The Newcastle-Ottawa scale (NOS) proposed by Well and colleagues was used to assess the methodological quality for cohort studies [7].

### Statistical analysis

In the analysis, random-effects meta-analysis was used to pool the association between hormone replacement therapy and risk of colorectal cancer mortality and the risk of all-cause mortality in patients with colorectal cancer diagnosed. We assessed methodological and clinical heterogeneity by *I*^*2*^ statistic, which quantifies the percentage of total variation across study results that could be explained by between-study heterogeneity rather than chance [8]. In this study, the cut-off value of low, moderate, and high heterogeneity were defined as an *I*^*2*^ statistic of 25, 50, and 75%, respectively [9]. As a separate analysis, the prediction interval, which describes the heterogeneity in a random-effects meta-analysis, was calculated to inform the variability of future treatment effects in 95% of all populations [10]. Potential publication bias and small-study effects were explored by visual inspection of funnel plots. We did not perform further statistical analysis for funnel plot asymmetric because the statistical power is limited when number of studies is fewer than 10 [11]. Also, we assessed all the articles for potential information bias. Each article was evaluated for its approach of assessing HRT exposure, which can be categorized into two groups: (1) questionnaire assessment and (2) objective documents, such as drug register or pharmacy database. To further examine the potential impact of information bias on HR estimates, we performed meta-regression where we regressed log-transformed HRs of each study by HRT exposure assessment method (objective documents or questionnaire). To assess the robustness of primary analysis, sensitivity analysis was conducted by omitting one study at a time [12]. In additional sensitivity analysis, a fixed-effect model was used to combine the data for the outcome. In this study, unless *p* < 0.0001, exact *p* values are provided. We used STATA version 15.1 (College Station, TX, USA) to analyse data.

## Results

### Literature search

**Fig. 1:**
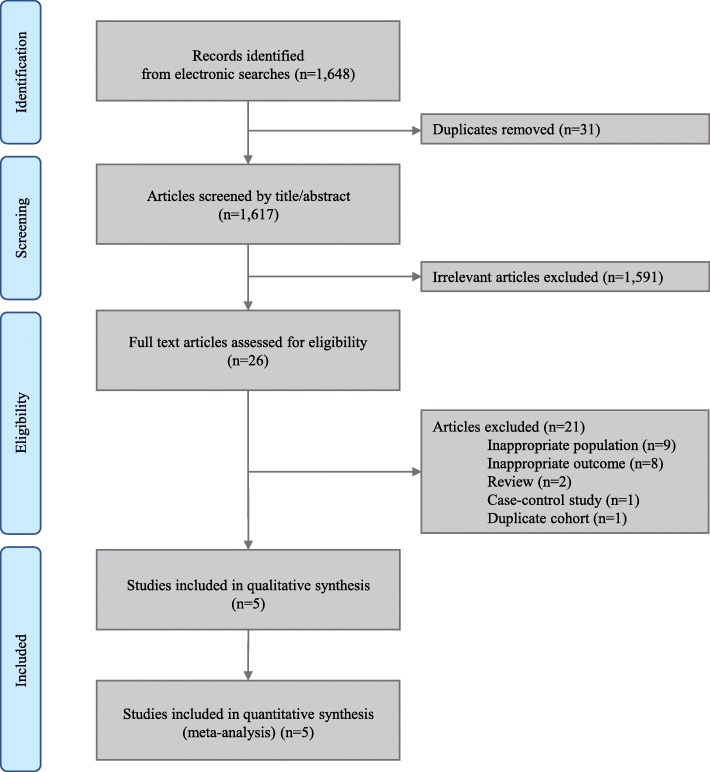
Study selection. Note. n: number

Figure 1 presents the literature search details and process. Briefly, we identified 1648 articles after initial literature searching and exclusion of the duplicated articles. We further rejected 1591 articles after reviewing titles and abstracts, because they were irrelevant to the scope of our analysis. For the 26 articles underwent full-text review, 17 were excluded because nine of them evaluated patients other than colorectal cancer survivors and eight reported results other than colorectal cancer-specific mortality or all-cause mortality. Two review articles, one case-control study, and one study reporting duplicate cohort were also excluded. Therefore, five unique studies met eligibility criteria. We did not identify additional article after reviewing the reference lists of eligible articles. Details on excluded studies are listed in the Additional file 1: Table S7.

### Characteristics of included studies

Overall, four prospective cohort studies [13–16] and one retrospective cohort study [17] were included in our meta-analysis, including relevant available data on current HRT user [13–17] and former HRT user [13, 14]. Table 1 presents the baseline characteristics of the included studies. The number of colorectal cancer survivors included in each study ranged from 699 to 5626. In total, we included 10,013 women for analysis. The studies were published from 1999 to 2018 and included patients from the United states [13–16] and Sweden [17]. Table S8 (See Additional file 1) shows the study quality assessed by Newcastle-Ottawa scale. The included studies are generally of low risk of bias, with Newcastle-Ottawa scale scores between seven and nine.

**Table 1:** Study characteristics of included studies

Study	Study period	Country	Age	Number of participants	Stage	Grade	HRT Type	HRT recency	CRC death/HRT user	All-cause death /HRT user	Mean follow-up year
Prospective cohort											
Arem et al. (2015) [13]	1995–2011	USA	50–71	2053	30.5% localized, 31.3% regional or distant, 38.2% unknown	12.0% well-differentiated, 57.5% moderate-differentiated, 0.9% undifferentiated, 29,6% unknown	E, E + P	Current, former	Current (98/670), former (36/201)	Current (214/670), former (87/201)	7.7
Chan et al. (2006) [14]	1976–2004	USA	62.2–65.7^a^	834	22.3% stage I, 26.1% stage II, 25.5% stage III, 15.6% stage IV, 10.5% unknown	22.3% stage I, 26.1% stage II, 25.5% stage III, 15.6% stage IV, 10.5% unknown	E, E + P	Current, former	Current (64/235), former (83/229)	Current (87/235), former (103/229)	5–10
Mandelson et al. (2003) [15]	1980–1998	USA	50–79	699	NR	NR	E, E + P	Current	Current (18/103)	Current (37/103)	5.33
Slattery et al. (1999) [16]	1991–1998	USA	55–79	801	35.4% local, 53.2% regional, 11.4% distant	NR	E, E + P	Current to stop less than 5 years	Current to stop less than 5 years (NR/186)	Current to stop less than 5 years (46/186)	4.0
Retrospective cohort											
Ji et al. (2018) [17]	2006–2015	Sweden	45–69	5626	23.7% stage I, 27.8% stage II, 36.2% stage III, 12.3% stage IV	NR	E, E + P	Current	Current (NR/1109)	Current (246/1109)	5.4
Study	Variables adjusted	Exposure assessment	Mortality ascertainment^b^								
Prospective cohort											
Arem et al. (2015) [13]	Years from questionnaire to diagnosis, BMI, marital status, smoking status, diabetes, physical activity, cancer stage, tumor grade, and receipt of chemotherapy, radiation, and surgery	Questionnaire	Social Security Administration Death Master File and the National Death Index Plus								
Chan et al. (2006) [14]	Age at diagnosis, year of diagnosis, cancer site (colon, rectum, or unknown), BMI, aspirin use at diagnosis, smoking status, postdiagnosis physical activity, cancer stage, tumor grade, and receipt of chemotherapy	Questionnaire	National Death Index and physician reviewer								
Mandelson et al. (2003) [15]	Age at diagnosis; year of diagnosis; and cancer stage	Group Health Cooperative computerized pharmacy database	Western Washington SEER registry								
Slattery et al. (1999) [16]	Age at diagnosis, study center, BMI, and cancer stage	In-person interview	Local tumor registries								
Retrospective cohort											
Ji et al. (2018) [17]	Age at diagnosis; year of diagnosis, country of birth (Sweden, European countries and others), highest educational level, family history of CRC, comorbidities with chronic ischemic heart disease, type 2 diabetes, chronic obstructive pulmonary disease and hypertension; and cancer stage	Swedish Prescribed Drug Register	Cause of Death Register								

^a^This study provided median age of postmenopausal participants. ^b^Causes of death and their corresponding *International Classification of Diseases and Related Health Problem, 9th and 10th version* (ICD-9 and ICD-10) codes were death due to colorectal cancer (ICD-9153, 154, and 159.0; and ICD-10 C18–C20, and C26), and death due to all the causes (ICD-9001–999 and ICD-10 A00–Z99). *HRT* hormone replacement therapy, *CRC* colorectal cancer, *E* estrogen therapy, *E + P* combined estrogen progesterone therapy, *NR* not reported, *BMI* body mass index, *SEER* Surveillance, Epidemiology, and End Results Program

### Meta-analysis

**Fig. 2:**
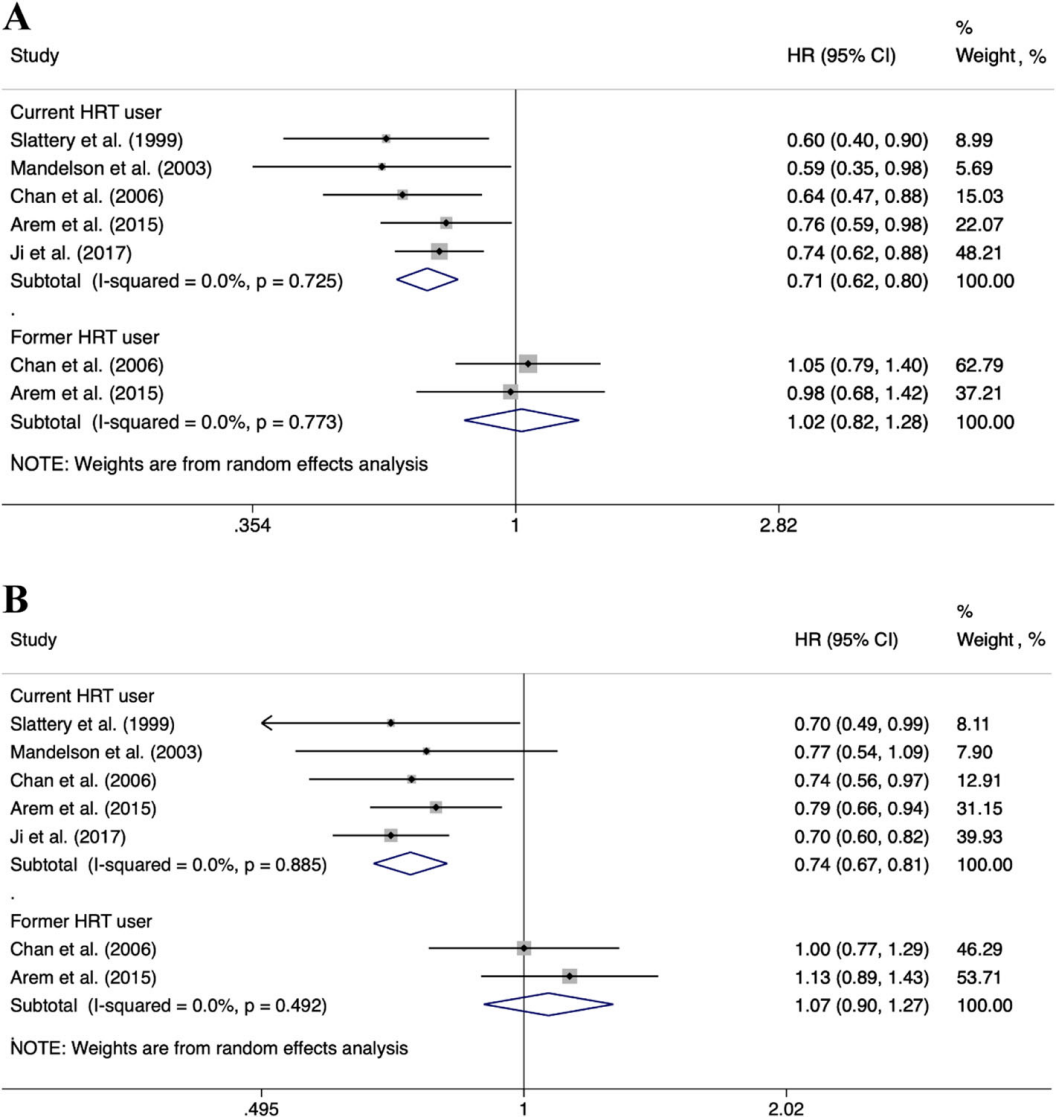
Summary of pooled risk estimates. The association between HRT use in women with colorectal cancer and (**a**) colorectal cancers and (**b**) all-cause mortality. Note. HR: hazard ratio, CI: confidence intervals, HRT: hormone replacement therapy

The meta-analysis results of pooling the five cohorts with 10,013 colorectal cancer survivors showed that the current use of HRT was associated with a significantly reduced risk of colorectal cancer mortality (HR, 0.71 [95% CI, 0.62–0.80], *I*^*2*^ = 0%) and all-cause mortality (HR, 0.74 [95% CI, 0.67–0.81], *I*^*2*^ = 0%) in survivors with colorectal cancer (Fig. 2). Low between-study variance is also reflected by the narrow prediction interval (colorectal cancer mortality, 0.58–0.86; and all-cause mortality, 0.63–0.87) (Additional file 1: Figures S1 and S2). The results of prediction interval indicated that, in a future study, colorectal cancer survivors with hormone replacement therapy are likely to have a reduced mortality risk compared with non-hormone users. On the other hand, among colorectal cancer survivors, former use of HRT was not significantly associated with reduced colorectal cancer-specific mortality (HR, 1.02 [95% CI, 0.82–1.28], *I*^*2*^ = 0%) and all-cause mortality (HR, 1.07 [95% CI, 0.90–1.27], *I*^*2*^ = 0%) (Fig. 2).

### Publication bias and information bias

We further assessed publication bias for studies evaluating the association between current use of HRT and mortality risks. Funnel plot asymmetry, which suggests the presence of publication bias and small-study effects, was not evident for included studies (Additional file 1: Figure S3 and S4). However, we did not perform statistical test to assess publication bias because limited number of eligible studies hampered the test power.

We examined the included studies for potential information bias. Among current users, the result showed no significant difference in pooled HRs of CRC-specific mortality for studies where HRT exposure was assessed by either questionnaire (HR 0.69 [95% CI, 0.57–0.82]) or objective document (HR 0.72 [95CI%, 0.61–0.85]) (*p* value for the difference = 0.6786). Similarly, no significant difference was observed between questionnaire assessment (HR 0.76 [95CI%, 0.67–0.87]) and objective document (HR 0.71 [95CI%, 0.62–0.82]) for all-cause mortality (*p* = 0.486).

### Sensitivity analysis

**Fig. 3:**
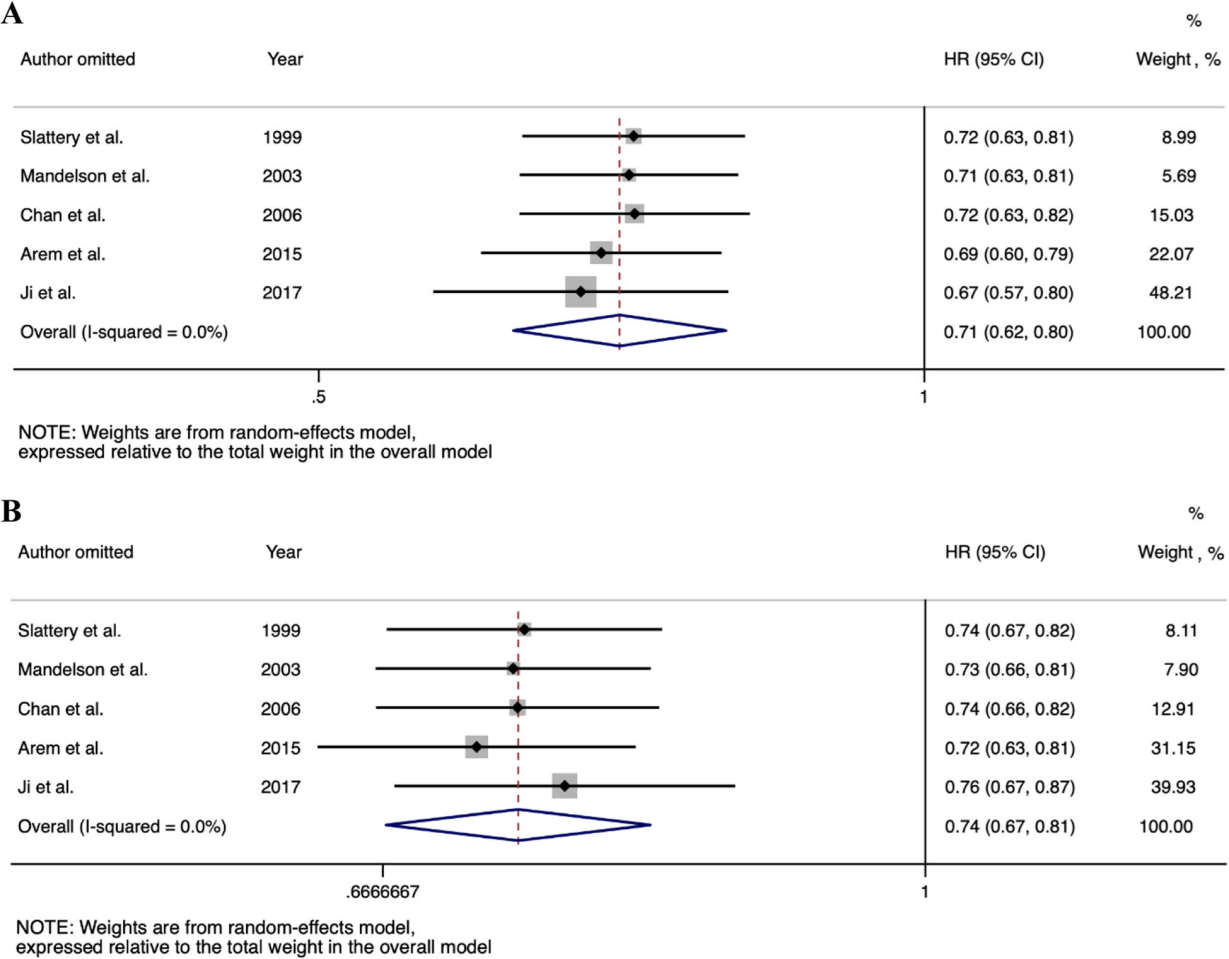
Sensitivity analysis. The association between current HRT use in women with colorectal cancer and (**a**) colorectal cancers and (**b**) all-cause mortality. Note. HR: hazard ratio, CI: confidence intervals, HRT: hormone replacement therapy

To assess the impact of individual studies on pooled estimates, sensitivity analysis was performed by omitting one study at a time. Figure 3 presents the pooled estimates after each study was omitted. Overall, no single study was identified to be substantially influential on the pooled estimates. For the current HRT users, omitting the most influential study by Ji et al. resulted in pooled HRs of 0.67 (0.57–0.80) for colorectal cancer mortality and 0.76 (0.67–0.87) for all-cause mortality, which were close to the primary analysis of 0.71 (0.62–0.80) for colorectal cancer mortality and 0.74 (0.67–0.81) for all-cause mortality. In a second sensitivity analysis, we re-ran the analysis with a fixed-effect meta-analysis. The effect estimates on colorectal cancer and all-cause mortality for current and former HRT users were unchanged (Additional file 1: Figure S5 and S6).

## Discussion

In this study, we performed a systematic review and meta-analysis, synthesising non-overlapping data from 10,013 colorectal cancer survivors, to quantify the association between HRT use and risk of mortality. The study findings show that the current use of HRT was associated with a significant reduction in the risk of colorectal cancer-specific mortality and all-cause mortality in women with colorectal cancer. In contrast, the former use of HRT was not associated with lower all-cause and colorectal cancer mortality in colorectal cancer survivors. Findings for the prediction interval were consistent with the main estimates. In sensitivity analysis, these results were not significantly affected in influence analysis. No differences were noted between two groups in the risk estimates using the fixed-effect meta-analysis.

Extensive evidence from clinical trials and observational studies have shown the use of HRT to be associated with a reduction in colorectal cancer risk in women [5]. In addition to the well-documented chemoprevention effects, our results also suggested a role of HRT on disease-specific and all-cause mortality among colorectal cancer survivors. There are potential mechanisms underlying the association between HRT and colorectal cancer and all-cause mortality in colorectal cancer survivors. As colorectal cancer may be a hormone-dependent cancer, tumor progression is inversely associated with expression of Estrogen Receptor Beta (ERß) [18, 19]. Konstantinopoulos and colleagues reported a significantly lower ERß expression in colon cancer cells compared with normal colon epithelium [20]. The reduction of ERß expression in colorectal cancer might be associated with loss of differentiation [20] and advanced cancer stages [21]. Furthermore, potential of colorectal cancer progression was reportedly repressed by ERß expression [19, 22]. Estrogen has been demonstrated to increase ERß expression [19]. The downstream genomic protective effects of estrogen result in gene transcription related to angiogenesis and cellular adhesion. In addition, ERß has been reported to induce apoptosis via different mechanisms, including increased p53 signalling in LoVo colon cancer cells and increased DNA fragmentation in COLO205 colon cancer cells [19]. In HT29 and SW480 colon cancer cells, ERß reduces cell proliferation via modulation of G1-phase cell cycle genes [23]. Non-genomic effects of estrogen interaction with ERß include activation of various intracellular signaling pathways [24]. Estrogenic regulation of c-Myc and cyclin D1 expression contributes to the inhibition of cell cycle progression [24]. Furthermore, selective activation of ERß has an anti-carcinogenic effect on tumor microenvironment via the downregulation of inflammatory signaling (interleukin-6) [19, 22].

To date, evidence is limited to determine whether the survival benefit of HRT among colorectal cancer survivors varies by dosage, duration, or timing of initiation. In our meta-analysis, among women diagnosed with colorectal cancer, the current HRT users had a reduced mortality risk compared with women with no prior hormone exposure, whereas risk was not changed for former users, suggesting that the association between HRT use and survivorship may be complicated and depend on the timing of hormone use. While evidence suggests that the current use of HRT has a survival benefit for colorectal cancer survivors, further rigorous assessment is needed to determine whether its possible adverse events such as venous thromboembolism outweighs the benefits. Because colorectal cancer is the third most common cancer in women [1], affecting 1 in 41 women over their lifetime period [25], our findings suggested that the potential favourable effects of HRT on mortality among colorectal cancer survivors justify further investigations.

### Strength

To our knowledge, this is the first meta-analysis to assess the association between HRT and the risk of colorectal cancer-specific and all-cause mortality in colorectal cancer survivors. Prior reviews focused on the association between risk of colorectal cancer incidence and hormone therapy; however, our analysis provided a clinically relevant insight to the impact of HRT use on survival of patients with colorectal cancer. Another important strength of this study is that we provide predictive interval to express between-study heterogeneity in a random-effects meta-analysis. We did not observe significant between-study variance, which was reflected by the narrow predictive interval (0.58–0.86) and (0.63–0.87) for colorectal cancer-specific and all-cause mortality among current HRT users, respectively. Accordingly, it is likely that a future study will show a reduced mortality risk in colorectal cancer survivors with hormone replacement therapy compared with non-hormone users.

### Limitation

There are several limitations that merit further discussion. First, owing to the study-level nature of the data and small numbers of studies, subgroup analyses and the assessment of publication bias were limited. Second, although we extensively searched for the best available evidence, studies identified in this analysis were from the United States and Sweden, which has limited the generalizability of the findings. However, we have provided prediction interval to provide the potential ranges of future studies. Third, meta-analyses of observational studies are susceptible to information bias. However, four of the five studies included were prospective design, which could minimize information bias. Also, in our meta-regression, no significant difference was observed between questionnaire assessment group or objective document group. Fourth, the mean follow-up period of included studies were relatively short, ranging from 4.0 to 7.7 years, which may be inadequate to reflect optimal long-term outcomes. Lastly, the ability to stratify the analyses by dose, duration, and types of hormone used was limited by the paucity of data, therefore, the pooled estimates represent a combined effect of estrogen and estrogen plus progesterone. Future research is needed to understand how these factors might influence survival of perimenopausal and postmenopausal women with a history of colorectal cancer.

## Conclusions

In this systematic review and meta-analysis, the current use of hormone replacement therapy was associated with a lower risk of colorectal cancer-specific mortality and all-cause mortality in colorectal cancer survivors. Further investigation is needed on the underlying mechanism to facilitate a personalized healthcare to inform cancer survivors.

## Supplementary information

**Additional file 1: Table S1.** Preferred Reporting Items for Systematic Reviews and Meta-Analyses (PRISMA) guidelines for the reporting of meta-analyses. **Table S2.** Search strategies on the PubMed. **Table S3.** Search strategies on the Ovid Embase. **Table S4.** Search strategies on the Cochrane library. **Table S5.** Search strategies on the Scopus. **Table S6.** Search strategies on the PsycINFO. **Table S7.** List of references with final exclusion reasons. **Table S8.** Newcastle-Ottawa Quality Assessment Scale for cohort studies. **Figure S1.** Prediction interval of colorectal cancer mortality for current users of hormone replacement therapy, using random-effects model. **Figure S2.** Prediction interval of all-cause mortality for current users of hormone replacement therapy, using random-effects model. **Figure S3.** Funnel plot of colorectal cancer mortality. **Figure S4.** Funnel plot of all-cause mortality. **Figure S5.** Forest plot of risk estimates of colorectal cancer mortality using the fixed-effect model. **Figure S6.** Forest plot of risk estimates of all-cause mortality using the fixed-effect model.

## Data Availability

All data generated or analysed during this study are included in this published article and its supplementary information files.

## Abbreviations

CI: Confidence intervals
ERß: Estrogen Receptor Beta
HR: Hazard ratio
HRT: Hormone replacement therapy
NOS: Newcastle-Ottawa scale
OR: Odds ratio
RR: Relative risk
