# Supplementary material for: Association of hormone replacement therapy with mortality in colorectal cancer survivor: a systematic review and meta-analysis
Source: BMC Cancer. 2019 Dec 9;19:1199. doi: 10.1186/s12885-019-6428-0 (PMC6902524; doi:10.1186/s12885-019-6428-0)
Supplement: Supplementary file 1 — Additional file 1: Table S1. Preferred Reporting Items for Systematic Reviews and Meta-Analyses (PRISMA) guidelines for the reporting of meta-analyses. Table S2. Search strategies on the PubMed. Table S3. Search strategies on the Ovid Embase. Table S4. Search strategies on the Cochrane library. Table S5. Search strategies on the Scopus. Table S6. Search strategies on the PsycINFO. Table S7. List of references with final exclusion reasons. Table S8. Newcastle-Ottawa Quality Assessment Scale for cohort studies. Figure S1. Prediction interval of colorectal cancer mortality for current users of hormone replacement therapy, using random-effects model. Figure S2. Prediction interval of all-cause mortality for current users of hormone replacement therapy, using random-effects model. Figure S3. Funnel plot of colorectal cancer mortality. Figure S4. Funnel plot of all-cause mortality. Figure S5. Forest plot of risk estimates of colorectal cancer mortality using the fixed-effect model. Figure S6. Forest plot of risk estimates of all-cause mortality using the fixed-effect model. [file 12885_2019_6428_MOESM1_ESM.docx]

**Table S1. Preferred Reporting Items for Systematic Reviews and Meta-Analyses (PRISMA) guidelines for the reporting of meta-analyses**

| **Section/topic** | **#** | **Checklist item** | **Reported on page #** |
| --- | --- | --- | --- |
| **TITLE** | | |  |
| Title | 1 | Identify the report as a systematic review, meta-analysis, or both. | Title |
| ABSTRACT | | |  |
| Structured summary | 2 | Provide a structured summary including, as applicable: background; objectives; data sources; study eligibility criteria, participants, and interventions; study appraisal and synthesis methods; results; limitations; conclusions and implications of key findings; systematic review registration number. | Abstract |
| **INTRODUCTION** | | |  |
| Rationale | 3 | Describe the rationale for the review in the context of what is already known. | Introduction, paragraph 1 and 2 |
| Objectives | 4 | Provide an explicit statement of questions being addressed with reference to participants, interventions, comparisons, outcomes, and study design (PICOS). | Introduction paragraph 3 |
| **METHODS** | | |  |
| Protocol and registration | 5 | Indicate if a review protocol exists, if and where it can be accessed (e.g., Web address), and, if available, provide registration information including registration number. | Methods, paragraph 1 |
| Eligibility criteria | 6 | Specify study characteristics (e.g., PICOS, length of follow-up) and report characteristics (e.g., years considered, language, publication status) used as criteria for eligibility, giving rationale. | Methods, paragraph 2 |
| Information sources | 7 | Describe all information sources (e.g., databases with dates of coverage, contact with study authors to identify additional studies) in the search and date last searched. | Methods, paragraph 1 |
| Search | 8 | Present full electronic search strategy for at least one database, including any limits used, such that it could be repeated. | Supplementary Table S2–S6 |
| Study selection | 9 | State the process for selecting studies (i.e., screening, eligibility, included in systematic review, and, if applicable, included in the meta-analysis). | Methods, paragraph 2 |
| Data collection process | 10 | Describe method of data extraction from reports (e.g., piloted forms, independently, in duplicate) and any processes for obtaining and confirming data from investigators. | Methods, paragraphs 3 |
| Data items | 11 | List and define all variables for which data were sought (e.g., PICOS, funding sources) and any assumptions and simplifications made. | Methods, paragraph 3 |
| Risk of bias in individual studies | 12 | Describe methods used for assessing risk of bias of individual studies (including specification of whether this was done at the study or outcome level), and how this information is to be used in any data synthesis. | Methods, paragraph 3 |
| Summary measures | 13 | State the principal summary measures (e.g., risk ratio, difference in means). | Methods, paragraph 4 |
| Synthesis of results | 14 | Describe the methods of handling data and combining results of studies, if done, including measures of consistency (e.g., *I^2^*) for each meta-analysis. | Methods, paragraph 4 |

**Table S2. Search strategies on the PubMed**

| #1 AND #2 AND #3 AND #4 (articles = 375) |
| --- |
| #1 Population: colorectal cancer survivor (articles = 706,413)  (cancer survivor [MeSH] OR cancer [MeSH] OR neoplasm [MeSH] OR carcinoma, colon [MeSH] OR carcinoma, colon [tw] OR carcinoma, rectal [MeSH] OR carcinoma, rectal [tw] OR carcinoma, colorectal [MeSH] OR carcinoma, colorectal [tw] OR colorectal neoplasm [MeSH] OR colorectal cancer [tw] OR colorectal malignan* [tw]) AND (surviv* [MeSH] OR surviv* [tw] OR prevoius* [tw] OR after [tw] OR recurren* [tw]) NOT (lung neoplasm [MeSH] OR carcinoma, non-small-cell lung [MeSH] OR carcinoma, non-small-cell lung [tw] OR non small cell lung cancer [tw] OR small cell lung cancer [tw] OR pulmonary malignancy [MeSH] OR pulmonary malignanc* [tw] OR lung adenocarcinoma [MeSH] OR lung adenocarcinoma [tw] OR lung squamous cell carcinoma [tw] OR lung large cell carcinoma [tw] OR head and neck neoplasm [MeSH] OR head and neck neoplasm [tw] OR esophageal neoplasm [MeSH] OR oesophageal cancer [tw] OR esophageal neoplasm [tw] OR liver neoplasm [MeSH] OR liver neoplasm [tw] OR liver cancer [tw] OR stomach neoplasm [MeSH] OR stomach neoplasm [tw] OR gastric cancer [tw] OR abdominal cancer [tw] OR abdominal carcinoma [tw] OR carcinomatosis [tw] OR head and neck malignan* [tw] OR oesophageal malignan* [tw] OR esophageal malignan* [tw] OR liver malignan* [tw] OR stomach malignan* [tw] OR gastric malignan* [tw] OR abdominal malignan* [tw] OR non-pulmonary malignan* [tw] OR hodgkin* disease [MeSH] OR hodgkin* disease OR lymphoma, non-hodgkin* [MeSH] OR lymphoma, non-hodgkin* OR leukemia [MeSH] OR leukemia [tw] OR haematopoietic cancer [MeSH] OR haematopoietic cancer* [tw] OR pyothorax* lymphoma [MeSH] OR pyothorax* lymphoma [tw] OR melanoma [tw] OR urothelial carcinoma [tw] OR urothelial cancer OR renal cell carcinoma [tw] OR melanoma [MeSH] OR urothelial carcinoma [MeSH] OR renal cell carcinoma [MeSH] OR breast cancer [MeSH] OR ovarian cancer [MeSH] OR uterine cancer [MeSH] OR cervical cancer [MeSH] OR endometrial cancer [MeSH] OR kidney and bladder cancer [MeSH] OR Prostate cancer [MeSH] OR breast cancer [tw] OR ovarian cancer [tw] OR uterine cancer [tw] OR cervical cancer [tw] OR endometrial cancer [tw] OR kidney and bladder cancer [tw] OR Prostate cancer [tw]) |
| #2 Exposure: hormone replacement therapy (articles = 309,728)  *estrogen [Mesh] OR *estrogen [tw] OR *estrogen replacement therapy [Mesh] OR *estrogen replacement therapy [tw] OR hormone replacement therapy [Mesh] OR hormone replacement therapy [tw] OR estradiol [Mesh] OR estradiol [tw] OR progestin therapy [Mesh] OR progestin therapy [tw] OR progestin [Mesh] OR progestin [tw] OR *progesterone [Mesh] OR *progesterone [tw] OR HRT [Mesh] OR HRT [tw] OR tibolone [Mesh] OR tibolone [tw] OR norethisterone [Mesh] OR norethisterone [tw] OR norethindrone [Mesh] OR norethindrone [tw] OR medrogestone [Mesh] OR medrogestone [tw] |
| #3 Outcome: colorectal cancer-specific mortality and all-cause mortality (articles = 3,502,242)  cause of death [MeSH] OR mortality [tw] OR mortalities [tw] OR death [tw] OR deaths[tw] OR fatality [tw] OR fatalities [tw] OR dying [tw] OR risk [MeSH] OR risk [tw] |
| #4 Study Design: RCTs, cohort, and case-control (articles = 2,023,323)  (randomised controlled trials [MeSH] OR cohort studies [MeSH] OR longitudinal studies [MeSH] OR follow up studies [MeSH] OR prospective studies [MeSH] OR retrospective studies [MeSH] OR survival analysis [MeSH] OR population-based stud* OR follow-up OR cohort [tw] OR longitudinal [tw] OR prospective [tw] OR retrospective [tw] OR incidence stud* [tw] OR incidence stud* [tw] OR concurrent stud* [tw] OR follow up [tw]) NOT (case control studies [MeSH] OR (case [tw] AND control [tw]) OR meta-analysis [MeSH]) |

**Table S3. Search strategies on the Ovid Embase**

| #1 AND #2 AND #3 AND #4 (articles = 1,021) |
| --- |
| #1 Population: colorectal cancer survivor (articles = 847,928)  (cancer surviv* OR cancer OR neoplasm OR carcinoma, colon OR carcinoma, rectal OR carcinoma, colorectal OR colorectal neoplasm OR colorectal cancer OR colorectal malignan*) AND (surviv* OR prevoius* OR after OR recurren*) NOT (lung neoplasm OR carcinoma, non-small-cell lung OR non small cell lung cancer OR small cell lung cancer OR pulmonary malignanc* OR lung adenocarcinoma OR lung squamous cell carcinoma OR lung large cell carcinoma OR head and neck neoplasm OR *esophageal neoplasm OR *esophageal cancer OR liver neoplasm OR liver cancer OR stomach neoplasm OR gastric cancer OR abdominal cancer OR abdominal carcinoma OR carcinomatosis OR head and neck malignan* OR *esophageal malignan* OR liver malignan* OR stomach malignan* OR gastric malignan* OR abdominal malignan* OR non-pulmonary malignan* OR hodgkin* disease OR hodgkin* disease OR lymphoma, non-hodgkin* OR lymphoma, non-hodgkin* OR leukemia OR leukemia OR haematopoietic cancer OR pyothorax* lymphoma OR melanoma OR urothelial carcinoma OR urothelial cancer OR renal cell carcinoma OR breast cancer OR ovarian cancer OR uterine cancer OR cervical cancer OR endometrial cancer OR kidney and bladder cancer OR prostate cancer) |
| #2 Exposure: hormone replacement therapy (articles = 177,084)  *estrogen OR *estrogen replacement therapy OR hormone replacement therapy OR estradiol OR progestin therapy OR progestin OR *progesterone OR HRT OR tibolone OR norethisterone OR norethindrone OR medrogestone |
| #3 Outcome: colorectal cancer-specific mortality and all-cause mortality (articles = 3,109,327)  cause of death OR mortality OR mortalities OR death OR deaths OR fatality OR fatalities OR dying OR risk |
| #4 Study Design: RCTs, cohort, and case-control (articles = 2,166,658)  (randomised controlled trials OR cohort studies OR longitudinal studies OR follow up studies OR prospective studies OR retrospective studies OR survival analysis OR population-based stud* OR follow-up OR cohort OR longitudinal OR prospective OR retrospective OR incidence stud* OR incidence stud* OR concurrent stud* OR follow up) NOT (case control studies OR meta-analysis) |

**Table S4. Search strategies on the Cochrane library**

| #1 AND #2 AND #3 AND #4 (articles = 4) |
| --- |
| #1 Population: colorectal cancer survivor (articles = 2,028)  (cancer surviv* OR cancer OR neoplasm OR carcinoma, colon OR carcinoma, rectal OR carcinoma, colorectal OR colorectal neoplasm OR colorectal cancer OR colorectal malignan*) AND (surviv* OR prevoius* OR after OR recurren*) NOT (lung neoplasm OR carcinoma, non-small-cell lung OR non small cell lung cancer OR small cell lung cancer OR pulmonary malignanc* OR lung adenocarcinoma OR lung squamous cell carcinoma OR lung large cell carcinoma OR head and neck neoplasm OR *esophageal neoplasm OR *esophageal cancer OR liver neoplasm OR liver cancer OR stomach neoplasm OR gastric cancer OR abdominal cancer OR abdominal carcinoma OR carcinomatosis OR head and neck malignan* OR *esophageal malignan* OR liver malignan* OR stomach malignan* OR gastric malignan* OR abdominal malignan* OR non-pulmonary malignan* OR hodgkin* disease OR hodgkin* disease OR lymphoma, non-hodgkin* OR lymphoma, non-hodgkin* OR leukemia OR leukemia OR haematopoietic cancer OR pyothorax* lymphoma OR melanoma OR urothelial carcinoma OR urothelial cancer OR renal cell carcinoma OR breast cancer OR ovarian cancer OR uterine cancer OR cervical cancer OR endometrial cancer OR kidney and bladder cancer OR prostate cancer) |
| #2 Exposure: hormone replacement therapy (articles = 188)  *estrogen OR *estrogen replacement therapy OR hormone replacement therapy OR estradiol OR progestin therapy OR progestin OR *progesterone OR HRT OR tibolone OR norethisterone OR norethindrone OR medrogestone |
| #3 Outcome: colorectal cancer-specific mortality and all-cause mortality (articles = 13,223)  cause of death OR mortality OR mortalities OR death OR deaths OR fatality OR fatalities OR dying OR risk |
| #4 Study Design: RCTs, cohort, and case-control (articles = 3,289)  (randomised controlled trials OR cohort studies OR longitudinal studies OR follow up studies OR prospective studies OR retrospective studies OR survival analysis OR population-based stud* OR follow-up OR cohort OR longitudinal OR prospective OR retrospective OR incidence stud* OR incidence stud* OR concurrent stud* OR follow up) NOT (case control studies OR meta-analysis) |

**Table S5. Search strategies on the Scopus**

| #1 AND #2 AND #3 AND #4 (articles = 223) |
| --- |
| #1 Population: colorectal cancer survivor (articles = 273,843)  (TITLE-ABS-KEY((cancer OR carcinoma OR colon OR rectal OR colorectal OR neoplasm OR malignan*) AND (surviv* OR previous))) AND NOT ((TITLE-ABS-KEY(lung OR "head and neck" OR *esophageal* OR liver OR stomach OR abdominal OR "non-pulmonary" OR hodgkin* OR lymphoma OR leukaemia OR "haematopoietic cancer")) OR (TITLE-ABS-KEY(pyothorax* OR lymphoma OR melanoma OR urothelial OR "renal cell carcinoma" OR breast OR ovarian OR uterine OR cervical OR endometrial OR "kidney and bladder" OR prostate))) |
| #2 Exposure: hormone replacement therapy (articles = 451,801)  TITLE-ABS-KEY(*estrogen OR "*estrogen replacement therapy" OR "hormone replacement therapy" OR estradiol OR "progestin therapy" OR progestin OR *progesterone OR HRT OR tibolone OR norethisterone OR norethindrone OR medrogestone) |
| #3 Outcome: colorectal cancer-specific mortality and all-cause mortality (articles = 5,478,408)  TITLE-ABS-KEY("cause of death" OR mortality OR mortalities OR death OR deaths OR fatality OR fatalities OR dying OR risk) |
| #4 Study Design: RCTs, cohort, and case-control (articles = 2,683,102)  (TITLE-ABS-KEY("randomi?ed controlled trials" OR "cohort stud*" OR "longitudinal stud*" OR "follow up stud*" OR "prospective stud*" OR "retrospective stud*" OR "survival analysis" OR "population-based stud*")) AND NOT (TITLE-ABS-KEY("case control stud*" OR "meta-analysis")) |

**Table S6. Search strategies on the PsycINFO**

| #1 AND #2 AND #3 AND #4 (articles = 25) |
| --- |
| #1 Population: colorectal cancer survivor (articles = 18,565)  (cancer surviv* OR cancer OR neoplasm OR carcinoma, colon OR carcinoma, rectal OR carcinoma, colorectal OR colorectal neoplasm OR colorectal cancer OR colorectal malignan*) AND (surviv* OR prevoius* OR after OR recurren*) NOT (lung neoplasm OR carcinoma, non-small-cell lung OR non small cell lung cancer OR small cell lung cancer OR pulmonary malignanc* OR lung adenocarcinoma OR lung squamous cell carcinoma OR lung large cell carcinoma OR head and neck neoplasm OR *esophageal neoplasm OR *esophageal cancer OR liver neoplasm OR liver cancer OR stomach neoplasm OR gastric cancer OR abdominal cancer OR abdominal carcinoma OR carcinomatosis OR head and neck malignan* OR *esophageal malignan* OR liver malignan* OR stomach malignan* OR gastric malignan* OR abdominal malignan* OR non-pulmonary malignan* OR hodgkin* disease OR hodgkin* disease OR lymphoma, non-hodgkin* OR lymphoma, non-hodgkin* OR leukemia OR leukemia OR haematopoietic cancer OR pyothorax* lymphoma OR melanoma OR urothelial carcinoma OR urothelial cancer OR renal cell carcinoma OR breast cancer OR ovarian cancer OR uterine cancer OR cervical cancer OR endometrial cancer OR kidney and bladder cancer OR prostate cancer) |
| #2 Exposure: hormone replacement therapy (articles = 10,398)  *estrogen OR *estrogen replacement therapy OR hormone replacement therapy OR estradiol OR progestin therapy OR progestin OR *progesterone OR HRT OR tibolone OR norethisterone OR norethindrone OR medrogestone |
| #3 Outcome: colorectal cancer-specific mortality and all-cause mortality (articles = 452,495)  cause of death OR mortality OR mortalities OR death OR deaths OR fatality OR fatalities OR dying OR risk |
| #4 Study Design: RCTs, cohort, and case-control (articles = 356,749)  (randomised controlled trials OR cohort studies OR longitudinal studies OR follow up studies OR prospective studies OR retrospective studies OR survival analysis OR population-based stud* OR follow-up OR cohort OR longitudinal OR prospective OR retrospective OR incidence stud* OR incidence stud* OR concurrent stud* OR follow up) NOT (case control studies OR meta-analysis) |

**Table S7. List of references with final exclusion reasons**

| **Study** | **Reason of exclusion** |
| --- | --- |
| Kyrø C, Frederiksen K, Holm M, Nørskov NP, Knudsen KE, Overvad K, et al. Prediagnosis plasma concentrations of enterolactone and survival after colorectal cancer: the Danish Diet, Cancer and Health cohort. *Br J Nutr*. 2018:1–12. | Inappropriate population |
| Symer MM, Wong NZ, Abelson JS, Milsom JW, Yeo HL. Hormone Replacement Therapy and Colorectal Cancer Incidence and Mortality in the Prostate, Lung, Colorectal, and Ovarian Cancer Screening Trial. *Clin Colorectal Cancer*. 2018;**17**:e281–288. | Inappropriate population, not reporting cancer survivors as study population |
| Lavasani S, Chlebowski RT, Prentice RL, Kato I, Wactawski‐Wende J, Johnson KC, et al. Estrogen and colorectal cancer incidence and mortality. *Cancer*. 2015;**121**:3261–3271. | Inappropriate population, not reporting cancer survivors as study population |
| Murphy N, Strickler HD, Stanczyk FZ, Xue X, Wassertheil-Smoller S, Rohan TE, et al. A prospective evaluation of endogenous sex hormone levels and colorectal cancer risk in postmenopausal women. *J Natl Cancer Inst*. 2015;**107**. | Inappropriate outcome, report colorectal cancer risk |
| Slattery ML, Herrick JS, Mullany LE, Gertz J, Wolff RK. Improved survival among colon cancer patients with increased differentially expressed pathways. *BMC Med*. 2015;**13**:75. | Inappropriate population |
| Lin JH, Zhang SM, Rexrode KM, Manson JE, Chan AT, Wu K, et al. Association between sex hormones and colorectal cancer risk in men and women. *Clin Gastroenterol Hepatol*. 2013;**11**:419–424. | Inappropriate population |
| Barnes EL, Long MD. Colorectal cancer in women: hormone replacement therapy and chemoprevention. *Climacteric*. 2012;**15**:250–255. | Review |
| Lin KJ, Cheung WY, Lai JY, Giovannucci EL. The effect of estrogen vs. combined estrogen‐progestogen therapy on the risk of colorectal cancer. *Int J Cancer*. 2012;**130**:419–430. | Review |
| Simon MS, Chlebowski RT, Wactawski-Wende J, Johnson KC, Muskovitz A, Kato I, et al. Estrogen plus progestin and colorectal cancer incidence and mortality. *J Clin Oncol*. 2012;**30**:3983–3990. | Inappropriate population, not reporting cancer survivors as study population |
| Hildebrand JS, Jacobs EJ, Campbell PT, McCullough ML, Teras LR, Thun MJ, et al. Colorectal cancer incidence and postmenopausal hormone use by type, recency, and duration in cancer prevention study II. *Cancer Epidemiol Biomarkers Prev*. 2009;**18**:2835–2841. | Inappropriate outcome, report colorectal cancer risk |
| Newcomb PA, Chia VM, Hampton JM, Doria-Rose VP, Dietz AT. Hormone therapy in relation to survival from large bowel cancer. *Cancer Causes Control*. 2009;**20**:409–416. | Case-control study |
| Rennert G, Rennert HS, Pinchev M, Lavie O, Gruber SB. Use of hormone replacement therapy and the risk of colorectal cancer. *J Clin Oncol*. 2009;**27**:4542–4547. | Inappropriate outcome, report colorectal cancer risk |
| Ritenbaugh C, Stanford JL, Wu L, Shikany JM, Schoen RE, Stefanick ML, et al. Conjugated equine estrogens and colorectal cancer incidence and survival: the Women's Health Initiative randomized clinical trial. *Cancer Epidemiol Biomarkers Prev*. 2008;**17**:2609–2618. | Duplicate cohort |
| Kampman E, Potter JD, Slattery ML, Caan BJ, Edwards S. Hormone replacement therapy, reproductive history, and colon cancer: a multicenter, case-control study in the United States. *Cancer Causes Control*. 1997;**8**:146–158. | Inappropriate outcome, report colorectal cancer risk |
| Persson I, Yuen J, Bergkvist L, Schairer C. Cancer incidence and mortality in women receiving estrogen and estrogen‐progestin replacement therapy—long‐term follow‐up of a Swedish cohort. *Int J Cancer*. 1996;**67**:327–332. | Inappropriate population |
| Calle EE, Miracle-McMahill HL, Thun MJ, Heath Jr CW. Estrogen replacement therapy and risk of fatal colon cancer in a prospective cohort of postmenopausal women. *J Natl Cancer Inst*. 1995;**87**:517–523. | Inappropriate outcome, report colorectal cancer risk |
| Newcomb PA, Storer BE. Postmenopausal hormone use and risk of large-bowel cancer. *J Natl Cancer Inst*. 1995;**87**:1067–1071. | Inappropriate outcome, report colorectal cancer risk |
| Jacobs EJ, White E, Weiss NS. Exogenous hormones, reproductive history, and colon cancer (Seattle, Washington, USA). *Cancer Causes Control*. 1994;**5**:359–366. | Inappropriate outcome, report colorectal cancer risk |
| Henderson BE, Paganini-Hill A, Ross RK. Decreased mortality in users of estrogen replacement therapy. *Arch Intern Med*. 1991;**151**:75–78. | Inappropriate population |
| Furner SE, Davis FG, Nelson RL, Haenszel W. A case-control study of large bowel cancer and hormone exposure in women. *Cancer Res*. 1989;**49**:4936–4940. | Inappropriate outcome, report colorectal cancer risk |
| Criqui MH, Suarez L, Barrett-Connor EL, McPhillips JA,  Wingard DL, Garland C. Postmenopausal estrogen use and mortality: results from a prospective study in a defined, homogeneous community. *Am J Epidemiol*. 1988;**128**:606–614. | Inappropriate population |

**Table S8. Newcastle-Ottawa Quality Assessment Scale for cohort studies**

| **Author** | **Year** | **Selection** |  |  |  | **Comparability** | **Outcome** |  |  | **Total stars** | **Risk of bias** |
| --- | --- | --- | --- | --- | --- | --- | --- | --- | --- | --- | --- |
|  |  | **Representativeness of the exposed cohort** | **Selection of the non-exposed cohort** | **Ascertainment of exposure** | **Demonstration that outcome of interest was not present at start of study** | **Comparability of cohorts on the basis of the design or analysis** | **Assessment of outcome** | **Was follow-up long enough for outcomes to occur** | **Adequacy of follow up of cohorts** |  |  |
| Arem et al. [13] | 2015 | * | * |  | * | ** | * | * | * | 8 | low |
| Chan et al. [14] | 2006 |  | * |  | * | ** | * | * | * | 7 | medium |
| Mandelson et al. [15] | 2003 | * | * | * | * | ** | * | * | * | 9 | low |
| Slattery et al. [16] | 1999 | * | * |  | * | ** | * | * | * | 8 | low |
| Ji et al. [17] | 2018 | * | * | * | * | ** | * | * | * | 9 | low |

Note. Studies awarded three or four stars for selection, two for comparability, and two or three for ascertainment of the outcome were defined to have low risk of bias. Medium risk of bias defined as those studies that rewarded two stars for selection, and one or two stars for comparability, and two or three for ascertainment of the outcome. Studies with zero or one stars for selection, or zero stars for comparability, or zero or one stars for ascertainment of the outcome were defined to have high risk of bias.

| 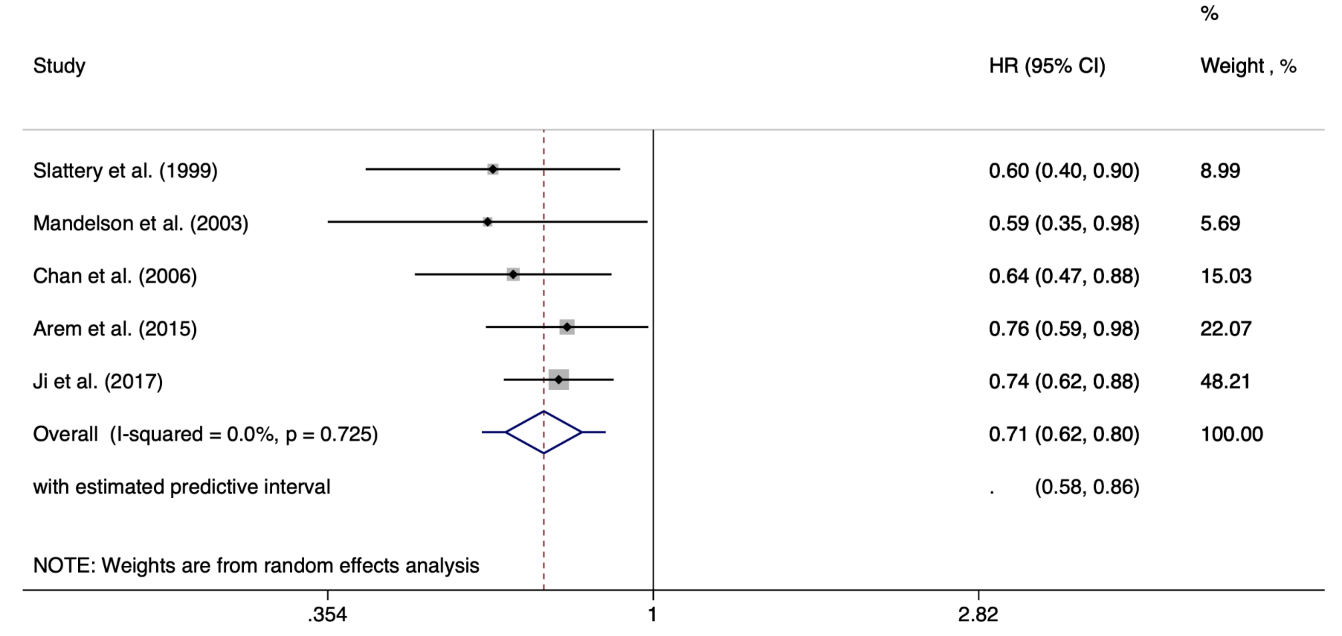 |
| --- |

**Figure S1. Prediction interval of colorectal cancer mortality for current users of hormone replacement therapy, using random-effects model**

Note. HR: hazard ratio, CI: confidence intervals.

| 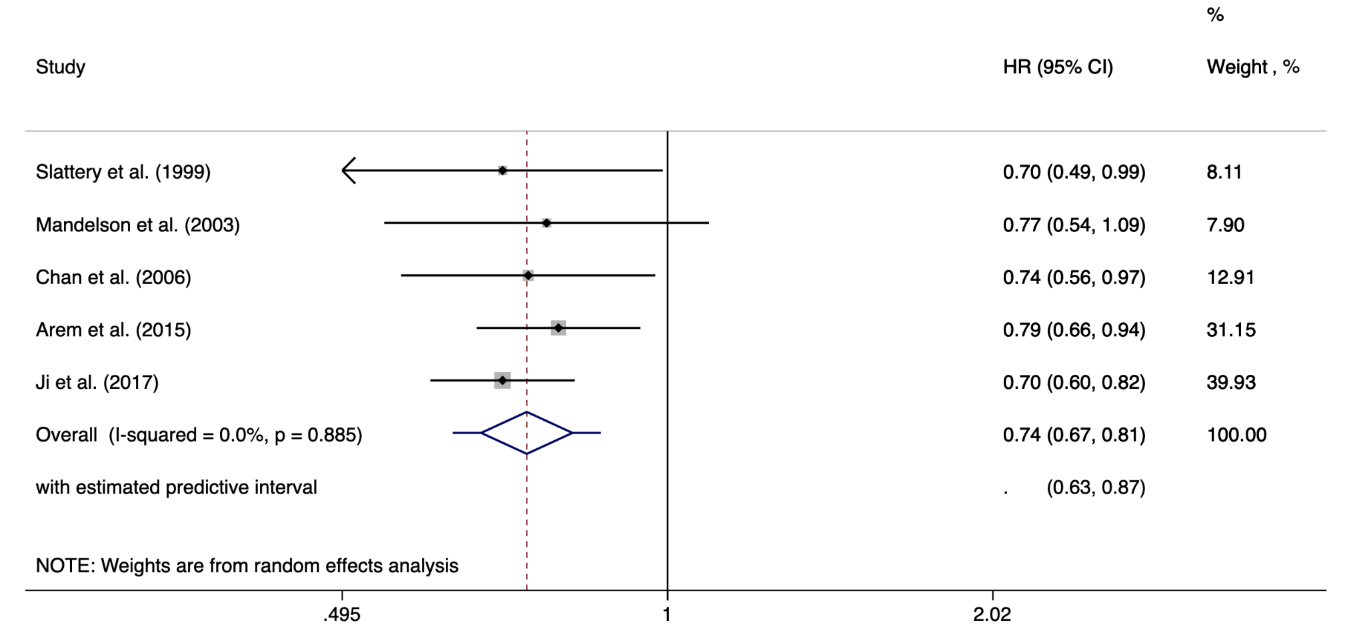 |
| --- |

**Figure S2. Prediction interval of all-cause mortality for current users of hormone replacement therapy, using random-effects model**

Note. HR: hazard ratio, CI: confidence intervals.

| 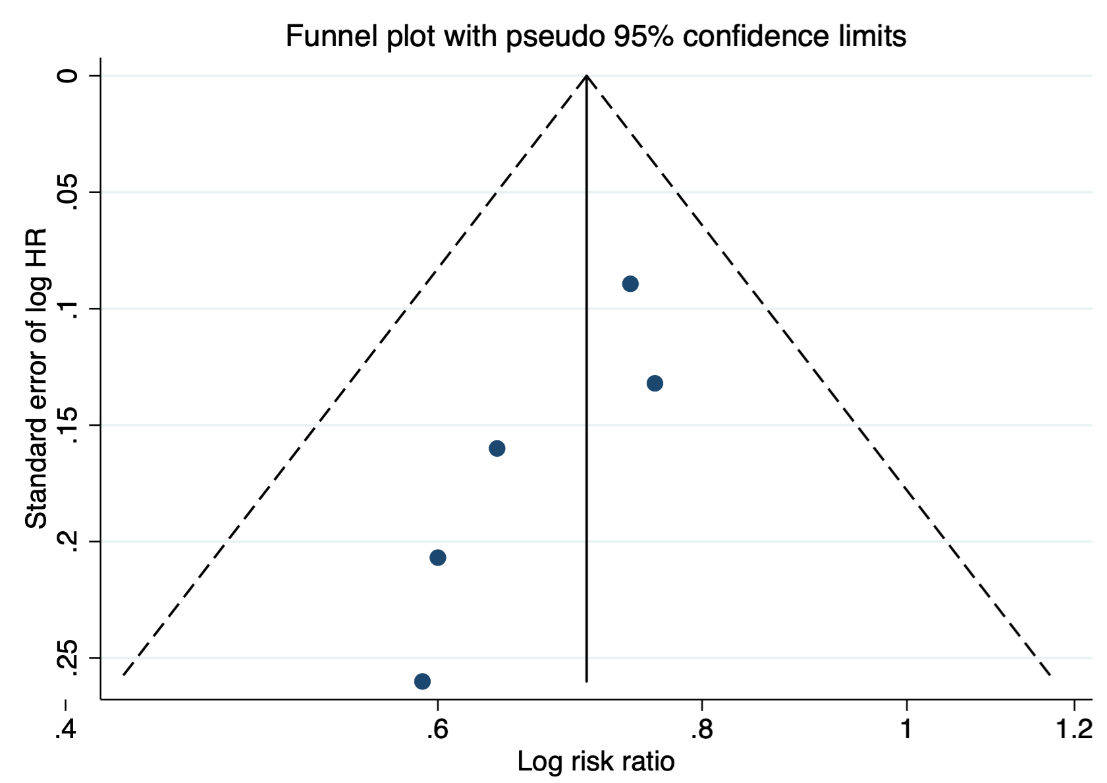 |
| --- |

**Figure S3. Funnel plot of colorectal cancer mortality**

Note. HR: hazard ratio.

| 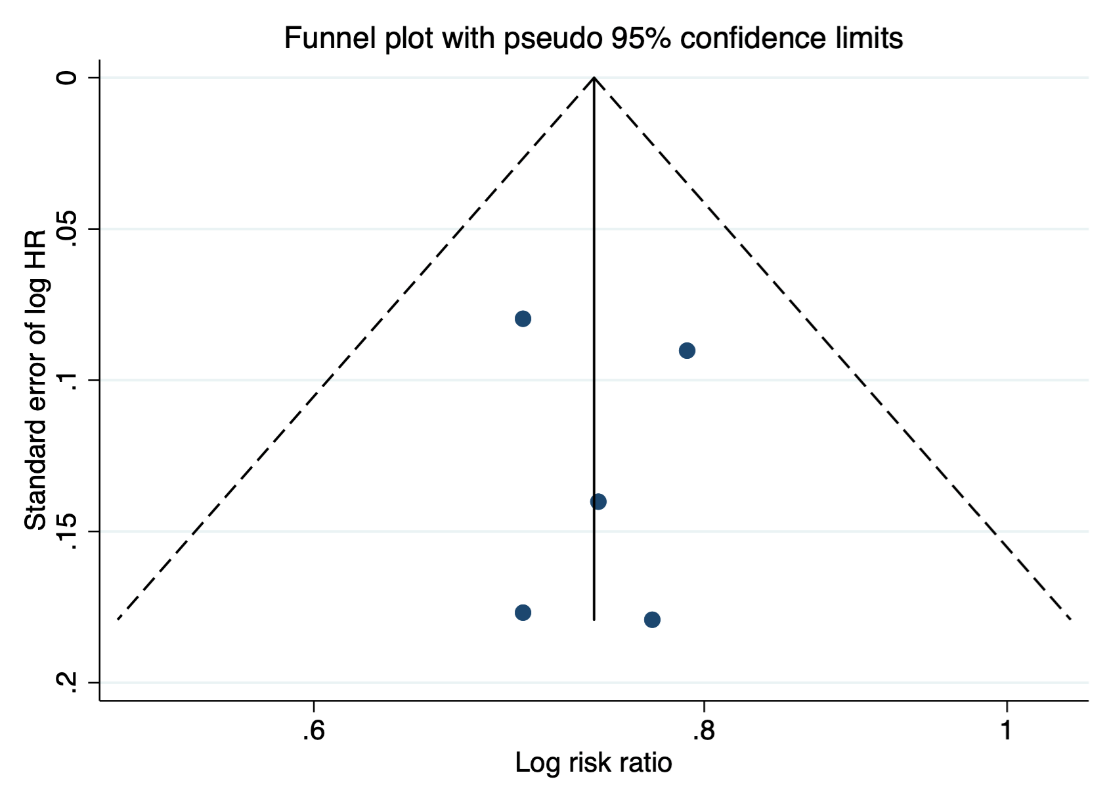 |
| --- |

**Figure S4. Funnel plot of all-cause mortality**

Note. HR: hazard ratio.

| 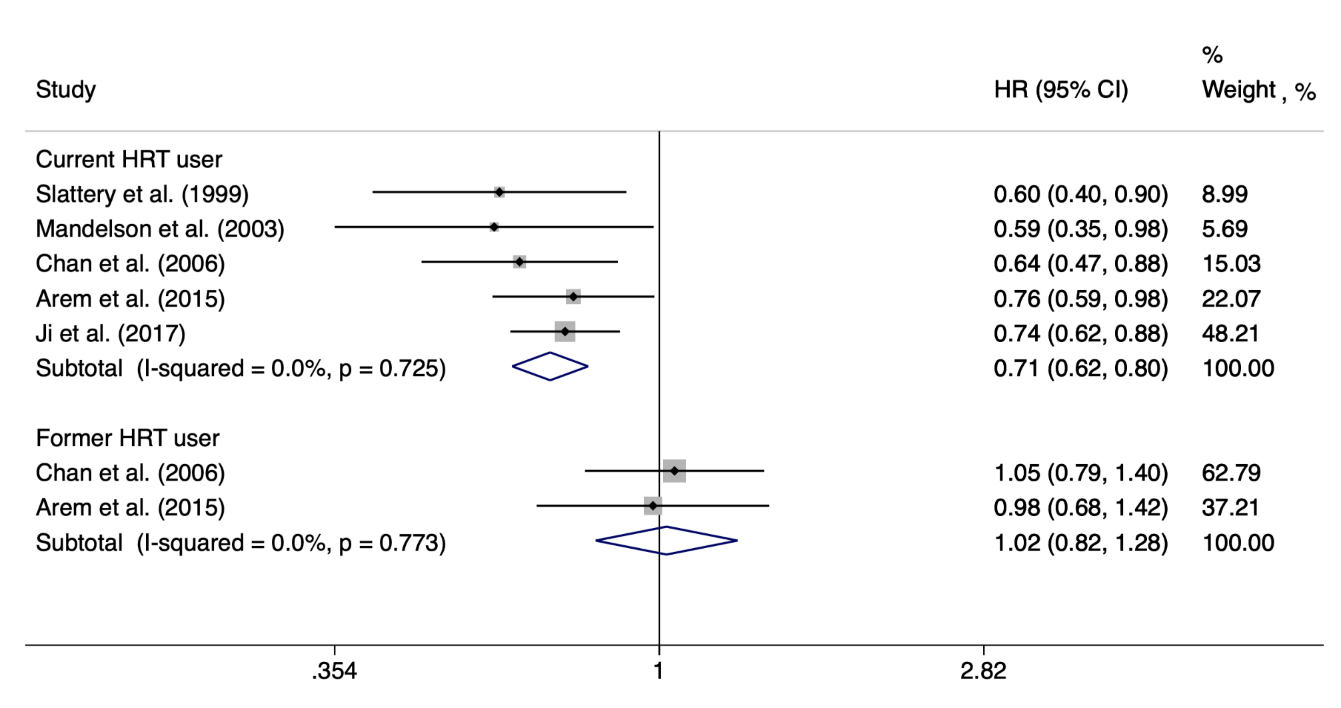 |
| --- |

**Figure S5. Forest plot of risk estimates of colorectal cancer mortality using the fixed-effect model**

Note. HR: hazard ratio, CI: confidence intervals, HRT: hormone replacement therapy.

| 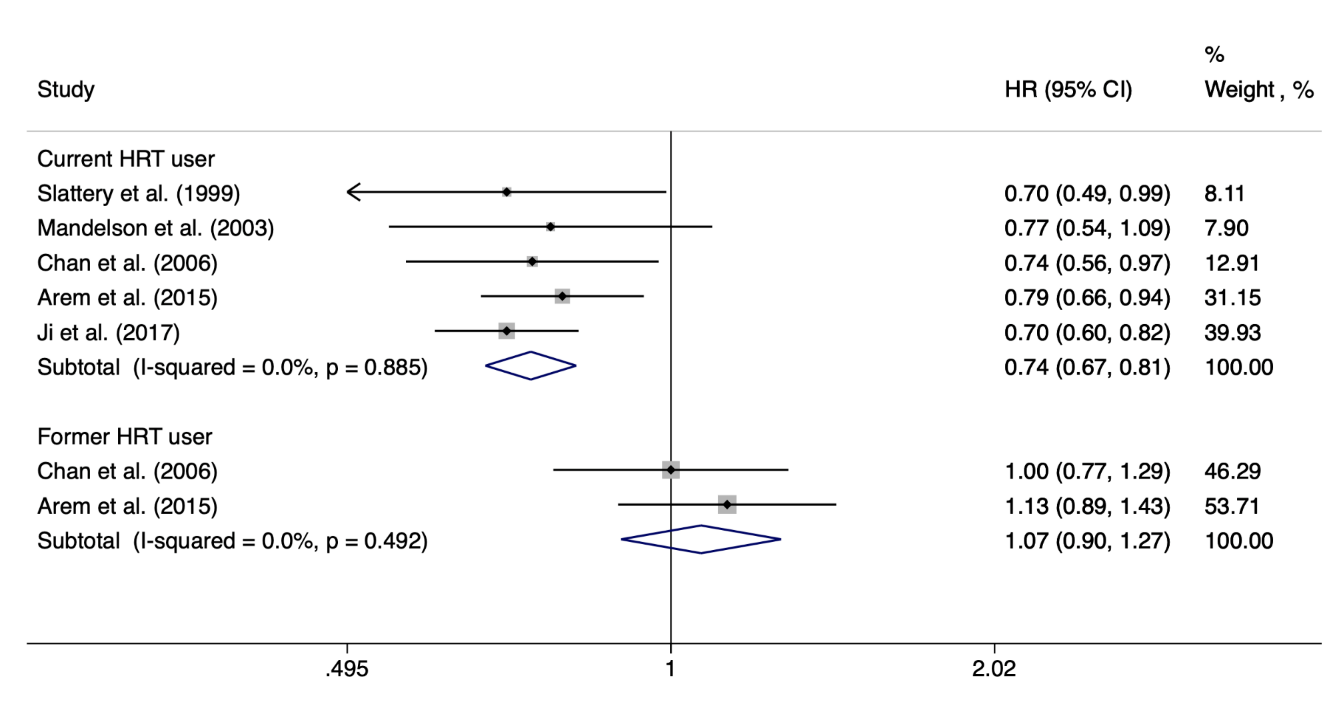 |
| --- |

**Figure S6. Forest plot of risk estimates of all-cause mortality using the fixed-effect model**

Note. HR: hazard ratio, CI: confidence intervals, HRT: hormone replacement therapy.
